# Association of lifestyle, physiological factors, and body composition with the facial skin microbiota in acne vulgaris

**DOI:** 10.3389/fmed.2025.1679925

**Published:** 2026-01-09

**Authors:** JeongHoon Lee, HyunChan Song, Ki-Young Kim

**Affiliations:** 1 BOGU Korean Medicine Clinic, Bucheon-si, Gyeonggi-do, Republic of Korea; 2 Graduate School of Biotechnology, Kyung Hee University, Seocheon/Yongin, Gyeonggi-do, Republic of Korea

**Keywords:** acne, *C. acnes*, microbiota, *S. aureus*, *S. epidermidis*, skin

## Abstract

**Introduction:**

The skin microbiota plays a crucial role in maintaining skin health and overall well-being. The composition of this microbial community is influenced by various host factors, including lifestyle habits, physiological parameters, and body composition.

**Methods:**

1,053 participants were included in this study. The composition of the skin microbiota was determined by analysing facial skin microbiome collection, obtained after the consent of participants. Also, physical characteristics of each participant were evaluated using answers from questionnaire. Potential microorganisms that contribute to acne vulgaris development were investigated. Statistical analysis was then performed based on the characteristics of the patient and normal groups, and the differences in the bacterial ratio grade assigned to each individual.

**Results:**

*C. acnes, S. aureus*, and *S. epidermidis* were significantly correlated with acne vulgaris. Several characteristics of the participants were closely correlated with the composition of the skin microbiota. There was significant differences among the participants' characteristics.

**Discussion:**

By analyzing the body composition and daily life of the study subjects, we identify associations of acne vulgaris and suggest specific lifestyle modifications that may be beneficial for acne sufferers.

## Graphical Abstract

**Figure:**
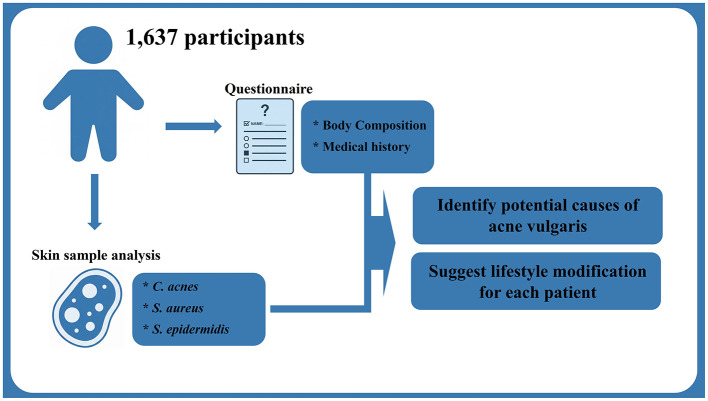

## 1 Introduction

Acne vulgaris is a common skin disease that involves several causes, including sebum production, inflammation, and the presence of bacteria (1–3). The disease affected 231 million people worldwide in 2019, resulting economic hardship for patients and straining healthcare systems worldwide (4). Previous studies have cited several factors as the main causes. However, because the skin's microbial ecosystem significantly influences various aspects of skin physiology, including sebum production and inflammation, the proportion of each type of bacteria on patients' skin has been studied (5–7). *Cutibacterium acnes* (formerly *Propionibacterium acnes*) was once thought to be the primary cause of acne, but it is now recognized as part of a broader microbial community that influences the onset and severity of acne (8). In these communities, it is known that certain bacteria, such as *C. acnes, Staphylococcus aureus*, and *Staphylococcus epidermidis*, are more likely to cause the condition.

These bacteria live in lipid-rich areas of the skin, where they can cause inflammation and trigger acne vulgaris (9, 10). However, their presence is not the only cause of acne. They play a variety of roles in maintaining skin health and balance (11).

In particular, *C. acnes* is found in abundance in sebum-rich areas (12). While once primarily known as a major cause of acne, recent studies suggests it also plays a variety of roles in regulating immune responses and balancing the skin (13, 14). Similarly, *S. aureus* is an opportunistic pathogen that causes several skin diseases, whereas *S. epidermidis* is now recognized as a beneficial bacterium that protects the skin by suppressing the growth of harmful bacteria (15, 16). These two bacteria interact in complex ways on the skin, influencing immune response and their living environment (17, 18).

The composition and activity of this microbial community are not determined not only by genetic factors. The physical characteristics and lifestyle of a host also have a significant impact (19). Factors such as sex, body mass index, sun exposure, hygiene practices, cosmetics, and even exercise habits can shape the microbial environment of our skin (20). Additionally, current health status of an individual can influence their skin microbiota (21). Therefore, it is important to understand how these individual factors contribute to changes in the skin microbiome and how these changes impact skin health.

In this study, we aimed to explore the complex relationship between acne vulgaris and the skin microbiome, and identify factors that may influence changes in the skin microbiota, including various physical characteristics and daily lifestyle habits.

## 2 Materials and methods

### 2.1 Study population

The study protocol received approval from the Kyung Hee University Institutional Review Board (IRB number: KHGIRB-24-540). Initial recruitment for the 2023–2024 skin measurement project involved 1,637 individuals from the BOGU Korean Medicine Clinic in Bucheon, South Korea (Table 1). The study participants were evenly distributed in age, including those aged 70 years or older.

**Table 1:** Number of participants in this study.

**Category**	**Number**
**Total participants**	1,637
**Analyzed participants**	1,053
Male participants	369
Female participants	684
**Acne research participants**	113
Healthy participants	80
Acne patients	33

Of the 1,637 initially recruited individuals, 584 were excluded due to withdrawal of consent (*n* = 71) and missing data on key questionnaires (*n* = 513). Thus, a total of 1,053 participants remained in the study until its completion and were included in the full cohort analysis. All remaining participants (*n* = 1,053) provided written informed consent and completed questionnaires regarding their medical history, the details of which were subsequently verified by Korea Health Information Service (KHIS), and information about their daily life (Table 2). All participants also underwent physical examination using the body composition analyzer Inbody970 (InBody, Korea). Regarding menstruation status, a portion of the female participants (*n* = 409) agreed to answer questions on this topic. This variable was handled using an available case analysis for relevant subgroup comparisons.

**Table 2:** The questionnaire provided to participants.

**Category**	**Details/Questions/Options**
Medical history	□ Hypertension □ Hyperlipidemia □ Diabetes □ Hepatitis (B/C) □ Tuberculosis □ Rheumatoid Arthritis □ Degenerative Arthritis □ Cardiovascular disease (Angina/Arrhythmia/Coronary artery disease) □ Cerebrovascular disease (Cerebral hemorrhage/Cerebral infarction) □ Cancer □ Thyroid disease □ Please write any other diseases you have experienced: ____
Allergies	_____
Medication currently taking	_____
Surgery	_____
For women	Start date of last period: Year __ Month __ Day __ □ Do you have menstrual cramps□□ Have had them since menarche □ Started after age 20 □ No menstrual cramps Menopause: ____
Smoking	□ Yes ( ___ cigarettes per day) □ No
Alcohol consumption	□ Yes ( ___ bottles per week, Type: ___, ___ times per week) □ No
Exercise	___ hours per day, Type: ___, ___ times per week
Respiratory-related conditions	□ Dyspnea (Difficulty breathing) □ Body aches □ Have COVID-19 sequelae □ Have vaccine side effects
Which part of your body do you consider the biggest problem?	□ Chronic fatigue (dizziness, lethargy, lack of strength) □ Tinnitus/Hearing loss □ Immunity (frequent colds/body aches, skin rash, stomatitis) □ Lack of sleep (Insomnia) □ Vascular disease (migraine, hyperlipidemia, diabetes management) □ Excessive stress (palpitations, sleep disorder) □ Family history (autoimmune disease, chronic inflammatory disease, etc.) □ Aftereffects of a car accident (full-body pain, dizziness) □ Women's health issues (menopausal syndrome, PMS, PCOS, miscarriage, infertility) □ Spinal/Joint pain (disc, stenosis, knee, shoulder, joint disease) □ Digestive disorders (indigestion, IBS, loss of appetite, gurgling) □ It's hard to explain where, but I feel discomfort
Do you apply moisturizer (lotion/cream)?	□ It's fine even if I don't apply it □ Only apply on dry days □ Apply every morning □ Carry it with me and apply periodically
Do you get skin trouble when you first use a new brand of cosmetics or jewelry?	□ Never □ Almost never □ Often □ Always
Do you have melasma, pigmentation, or freckles on your face?	□ Not at all □ Almost none □ Have a few □ Have often/a lot
How much time do you spend on outdoor activities exposed to sunlight per day?	□ Less than 1 h □ 1–2 h □ 3–4 h □ 5 h or more
Have you ever been diagnosed with the following symptoms at a hospital?	□ Acne □ Atopy □ Eczema □ Itching (Pruritus) □ Hives (Urticaria) □ Psoriasis □ Melasma/Blemishes □ Cafe-au-lait spot □ Ota's nevus

**Figure 1:**
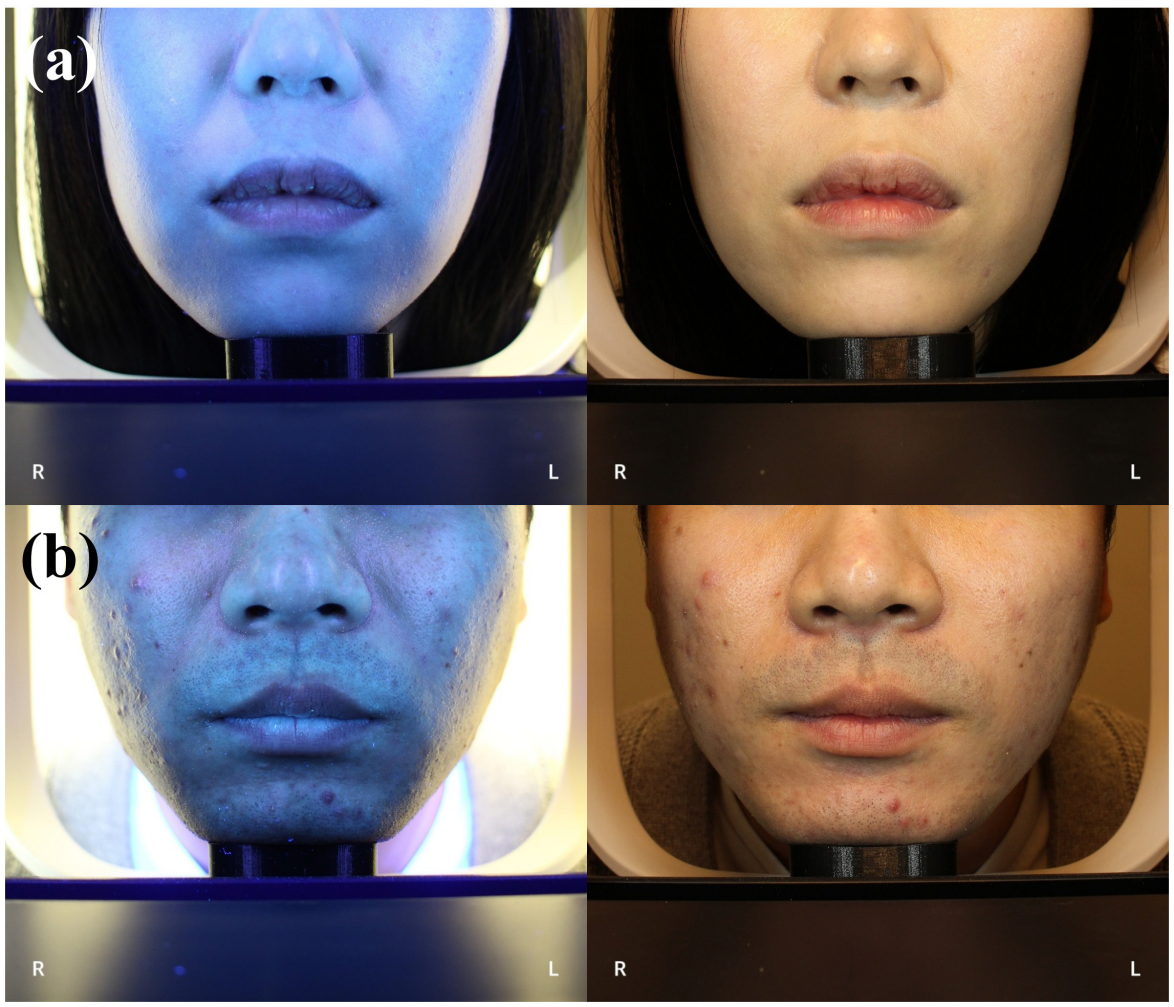
F-ray image of participants. **(a)** F-ray and normal image of a participant from normal group **(b)** F-ray and normal image of a participant with acne vulgaris.

From the full cohort (*n* = 1,053), a clinically phenotyped subset of 113 participants was further selected for F-ray imaging (22) and dedicated subgroup analyses (Figures 1a, b). This subset consisted of two distinct groups, an acne vulgaris patient group (*n* = 33) and a healthy control group (*n* = 80). The inclusion criterion for acne vulgaris patient group was defined by a clinical assessment of moderate or greater acne severity, specifically requiring a Global Acne Grading System (GAGS) score over 19 (23). Individuals were excluded if they had used systemic antibiotics within the 4 weeks prior to the study, had a current diagnosis of other chronic inflammatory skin diseases, or did not consent to participate in the specific acne vulgaris research. The healthy control group was confirmed to have no history of skin-related diseases, as verified by the hospital and KHIS records prior to the study.

### 2.2 Body composition calculation

Height (cm), weight (kg), skeletal muscle mass (kg), body fat mass (kg), body mass index (BMI) (kg/m^2^), body fat percentage (%), extracellular water (ECW) ratio, waist-to-hip ratio (WHR), skeletal muscle index (SMI) (kg/m^2^), and phase angle were measured using the body composition analyzer Inbody970 (InBody, Korea). Calculations were performed using the LookinBody 510 (InBody, Korea) program. The following equations that require calculation for each result were used (24–27).


$$\begin{array}{l} \text{BMI }(\text{kg/m})\;=\text{ Weight }(\text{kg})/(\text{Height }(\text{m})\;\times \text{ Height }(\text{m}))\;\\ \text{Body Fat Percentage }(\%)\;=\;\\ \;(\text{Body Fat Mass }(\text{kg})\text{/Weight }(\text{kg}))\;\times \;100\;\\ \text{ECW Ratio }=\;\\ \text{ Extracellular Water }(\text{ECW})\text{/Total Body Water }(\text{TBW})\;\\ \text{WHR }=\text{ Waist Circumference }(\text{cm})\text{/Hip Circumference }(\text{cm})\;\\ \text{SMI }=\text{ Skeletal Muscle Mass }(\text{kg})/(\text{Height }(\text{m})\;\times \text{ Height }(\text{m}))\; \end{array}$$

### 2.3 Skin sample collection

Skin samples were collected using sampling discs (D-Squame, USA, 22.0 mm). Two sampling discs were applied to each cheekbone of patients diagnosed with acne vulgaris, and sampling discs were attached to the same area of normal participants to serve as a control group using a D-Squame Disc Applicator (D-Squame, USA) at a pressure of 225 g/cm^2^ for 5 s (28). The discs were left in place for 10 min and placed in 2.0 mL microcentrifuge tubes (Eppendorf, USA). All samples collected from hospitals were stored in an A202W refrigerator (LG, Korea) before shipping. The samples were packed in Styrofoam boxes with dry ice and transported to the laboratory.

### 2.4 Microbiota analysis

Rather than analyzing the entire skin microbiome, this study focused on three specific microbial species (*C. acnes, S. aureus*, and *S. epidermidis*) known to be associated with acne vulgaris based on previous research (29, 30). This targeted approach was adopted as a strategic measure to efficiently manage a large sample of 1,053 people. Microbial RNA was purified using TRIzol (Ambion, USA). After purification, RNA was precipitated using isopropanol. Total RNA from each sample was used to synthesize cDNA using reverse transcriptase (Thermo Fisher Scientific, USA).

Real-time PCR was performed using the synthesized cDNA as a template using qPCR master mix (BioFACT, Korea). Primers were designed to target 16s rRNA of *C. acnes, S. aureus*, and *S. epidermidis* (Table 3). The suspension was heated at 95 °C for 5 min for initial denaturation. The PCR cycle consisted of 28 cycles of 95 °C for 20 s, 55 °C for 20 s, and 72 °C for 20 s. Real-time PCR was performed using a CFX Connect instrument (Bio-Rad, USA). Each qRT-PCR analysis was repeated three times to confirm the reproducibility of the measurements.

**Table 3:** Primers used in this study.

**Name**	**Sequence**
*C. acnes*_F	TAA CTT CAG GAA ACT GGG GC
*C. acnes*_R	TCA AAG CCT TGG TAA GCC AC
*S. aureus*_F	GAC GCT GAT GTG CGA AAG CG
*S. aureus*_R	CAA CCT TGC GGT CGT ACT CC
*S. epidermidis*_F	ACA GAC GAG GAG CTT GCT CC
*S. epidermidis*_R	TAC CAA CTA GCT AAT GCG GC

### 2.5 Standardization of real-time data

Real-time PCR results were expressed as Ct values, which represent the number of cycles required to produce a signal greater than the threshold. All Ct values were then converted to relative abundance, which represents the proportion of each bacterial species in the overall microbial community.

### 2.6 Statistical analysis

Descriptive statistics were used to summarize participant characteristics, including means and standard deviations (SD) for normally distributed continuous variables, or medians and interquartile ranges (IQR) for non-normally distributed variables. Frequencies and percentages were used for categorical variables.

Correlations between skin microbiota diversity and continuous variables were assessed using Pearson correlation coefficient (r) for normally distributed data and Spearman rank correlation coefficient (ρ) for non-normally distributed data.

Comparisons of population between two independent groups were performed using an Independent *t*-test for normal distribution, or a Mann-Whitney *U*-test for non-normal distribution. For comparisons among three or more groups, ANOVA followed by Tukey's *post-hoc* test, or the Kruskal-Wallis test followed by Dunn's *post-hoc* test, was used.

All statistical analyses were performed using GraphPad Prism version 8.0.2 (Graph Pad Software Inc., USA). *P-values* were derived from the specific statistical tests performed, and a *P* < 0.05 was considered statistically significant for all comparisons.

## 3 Results

### 3.1 Skin microbiota composition of acne vulgaris patients

**Figure 2:**
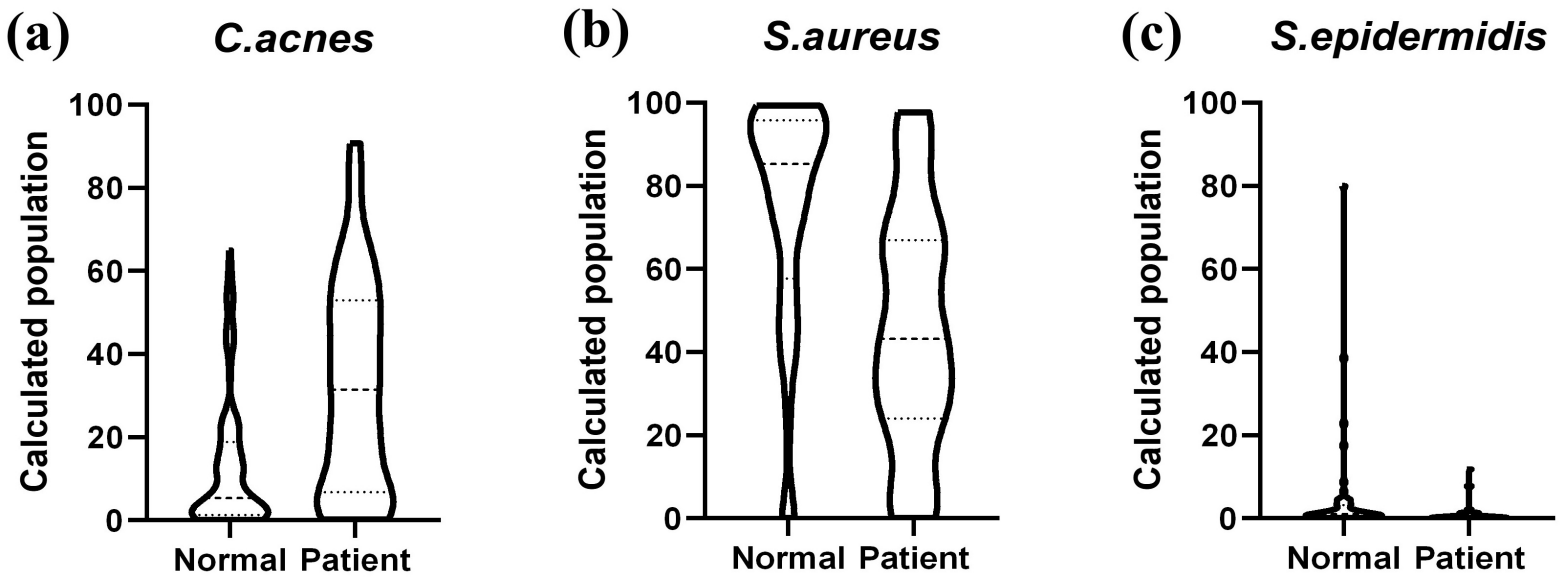
Comparison of relative abundance of *C. acnes, S. aureus*, and *S. epidermidis* in normal and patient group. **(a)** *C. acnes* abundance in normal and patient group. **(b)** *S. aureus* abundance in normal and patient group. **(c)** *S. epidermidis* abundance in normal and patient group.

The skin microbiota of acne vulgaris patients showed significant differences in the abundance of key microbial species compared to healthy controls (Figure 2). As expected, *C. acnes* was significantly more abundant in acne vulgaris patients, supporting the well-established role of *C. acnes* in acne vulgaris pathogenesis. However, the wide variation of *C. acnes* abundance in both groups suggests that factors other than bacterial abundance are responsible. In healthy participants, *S. aureus* was relatively more abundant and comprised a higher proportion of the skin microbiota, but its relative abundance was lower in acne vulgaris patients. Similarly, *S. epidermidis* was significantly more abundant in healthy individuals, but was nearly absent in acne vulgaris patients.

### 3.2 Skin microbiota composition according to daily lives

**Figure 3:**
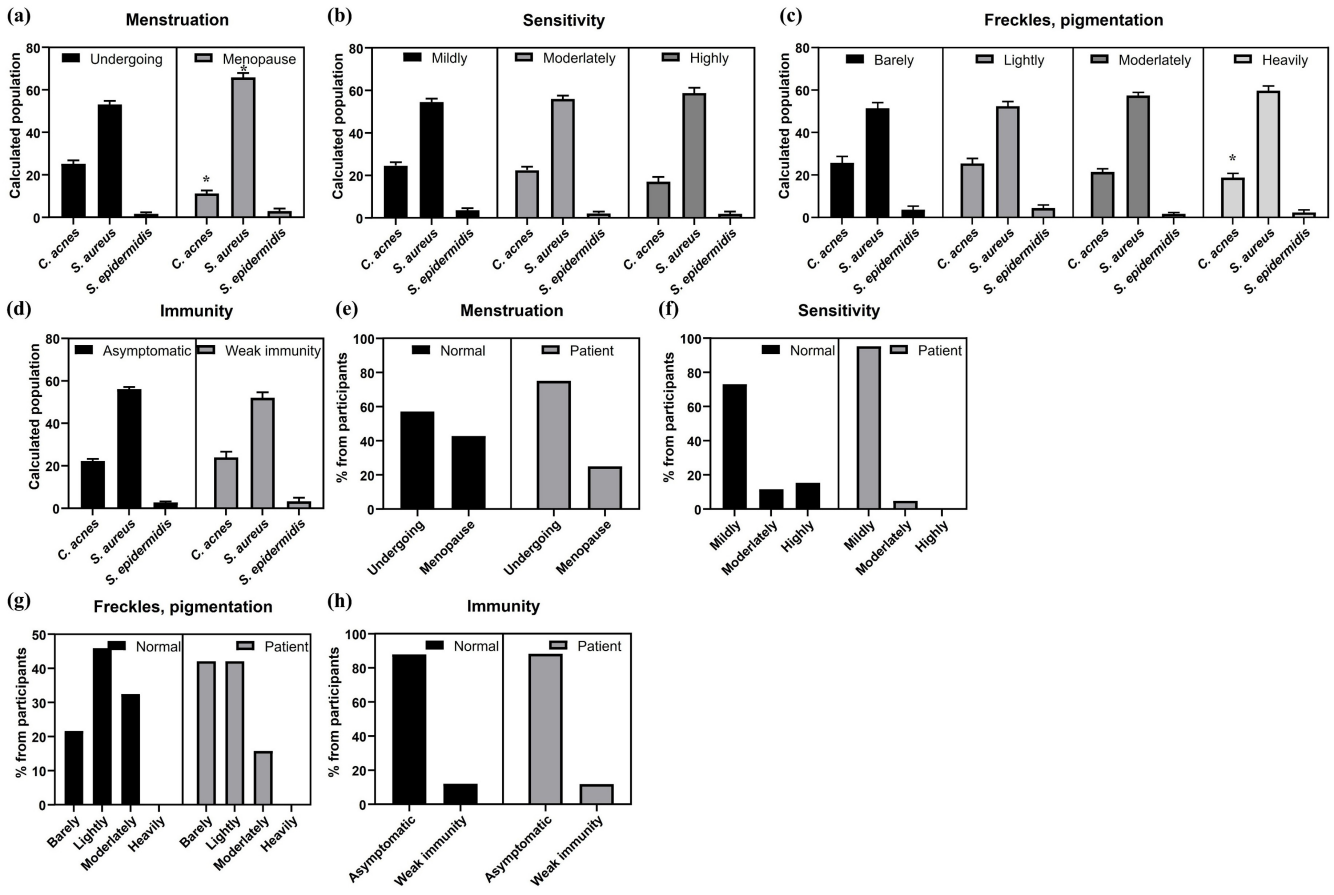
The analysis of the skin microbiota in different subgroups of participants based on their responses to the questionnaire and its comparison between the normal and patient groups. **(a)** Skin microbiota composition by smoking status. **(b)** Skin microbiota composition by exercise frequency. **(c)** Skin microbiota composition by sun exposure frequency. **(d)** Skin microbiota composition by moisturizer application frequency. **(e)** Smokers in acne vulgaris patients and normal group. **(f)** Exercise frequency in acne vulgaris patients and normal group. **(g)** Sun exposure frequency in acne vulgaris patients and normal group. **(h)** Applying moisturizer frequency in acne vulgaris patients and normal group. (*: *p*-value < 0.05).

To assess the influence of daily life, the relationship between lifestyle factors and skin microbiota was analyzed. Smoking slightly increased the abundance of *S. aureus* and decreased the number of *C. acnes*, suggesting a potential disruption of the microbiome, but the decrease was smaller than previously reported (Figure 3a). Exercise frequency did not significantly affect bacterial abundance (Figure 3b). Sun exposure significantly increased the abundance *S. epidermidis* and slightly decreased *S. aureus* (Figure 3c). Moisturizer use was not correlated with *C. acnes* or *S. aureus*, and *S. epidermidis* did not change consistently with moisturizing frequency (Figure 3d).

To evaluate the impact of lifestyle habits on skin microbiota, smoking, exercise, sun exposure, and moisturizer use were compared. The smoking rate was slightly higher in the patient group (9.52%) than the normal group (6.25%), but there was no significant difference (Figure 3e). No clear association was found between exercise frequency and acne vulgaris status, with the patient group showing a complex distribution pattern including both higher rates of daily exercise and infrequent exercise (Figure 3f). Both groups had similar sun exposure, but only the patient group reported moderate exposure (Figure 3g). Moisturizer use showed significant differences: patients used it occasionally and regularly (78.6% and 14.3%), while the normal group used it more evenly, and infrequent moisturizer use was lower in the patient group (Figure 3h).

### 3.3 Skin microbiota composition according to medical history

**Figure 4:**
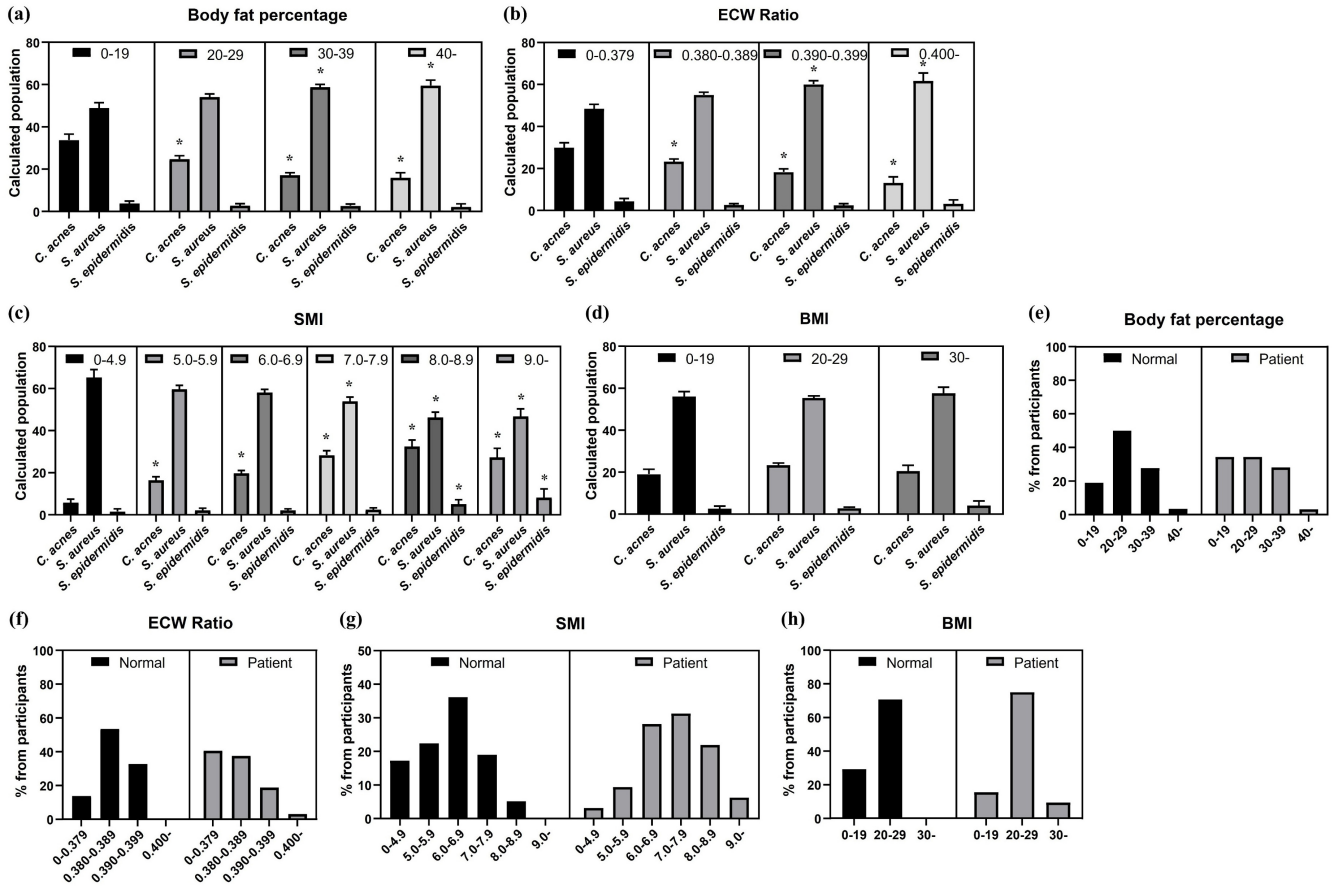
The analysis of the skin microbiota in different subgroups of participants based on their medical history its comparison between the normal and patient groups. **(a)** Skin microbiota composition by current menstruation (*n* = 409). **(b)** Skin microbiota composition by skin sensitivity. **(c)** Skin microbiota composition by presence of freckles and pigmentation. **(d)** Skin microbiota composition by immune disorder. **(e)** Menstrual status between acne vulgaris patients and normal group (*n* = 409). **(f)** Skin sensitivity between acne vulgaris patients and normal group. **(g)** Immune disorders between acne vulgaris patients and normal group. **(h)** Freckles and pigmentation between acne vulgaris patients andnormal group. (*: *p*-value < 0.05).

Analysis of the skin microbiota of all participants showed an association between bacterial abundance and clinical diagnosis. Menstruating individuals had increased abundance of *C. acnes*, whereas postmenopausal individuals had decreased abundance of *S. aureus* (Figure 4a). Skin sensitivity and the presence of skin freckles or pigmentation were weakly correlated with bacterial abundance (Figures 4b, c). Unexpectedly, as the degree of skin sensitivity or pigmentation increased, the abundance of *C. acnes* decreased, and the abundance of *S. aureus* increased. However, immunity did not show any significant association with the skin microbiota (Figure 4d).

Then, we statistically analyzed menstrual status, sensitivity, immunity and freckles/pigmentation to find out the differences between the acne vulgaris patient group and the normal group. Among all women who responded about menstrual status, 57.1% of the participants without acne vulgaris were menstruating, compared to 75.0% of patients (Figure 4e). Acne vulgaris patients reported less skin sensitivity and a lower degree of freckles or pigmentation compared to the control group (Figures 4f, h). However, immunity status did not show any difference between the patient group and the normal group, which was similar to the previous result (Figures 4d, g).

### 3.4 Skin microbiota composition according to body composition analysis

**Figure 5:**
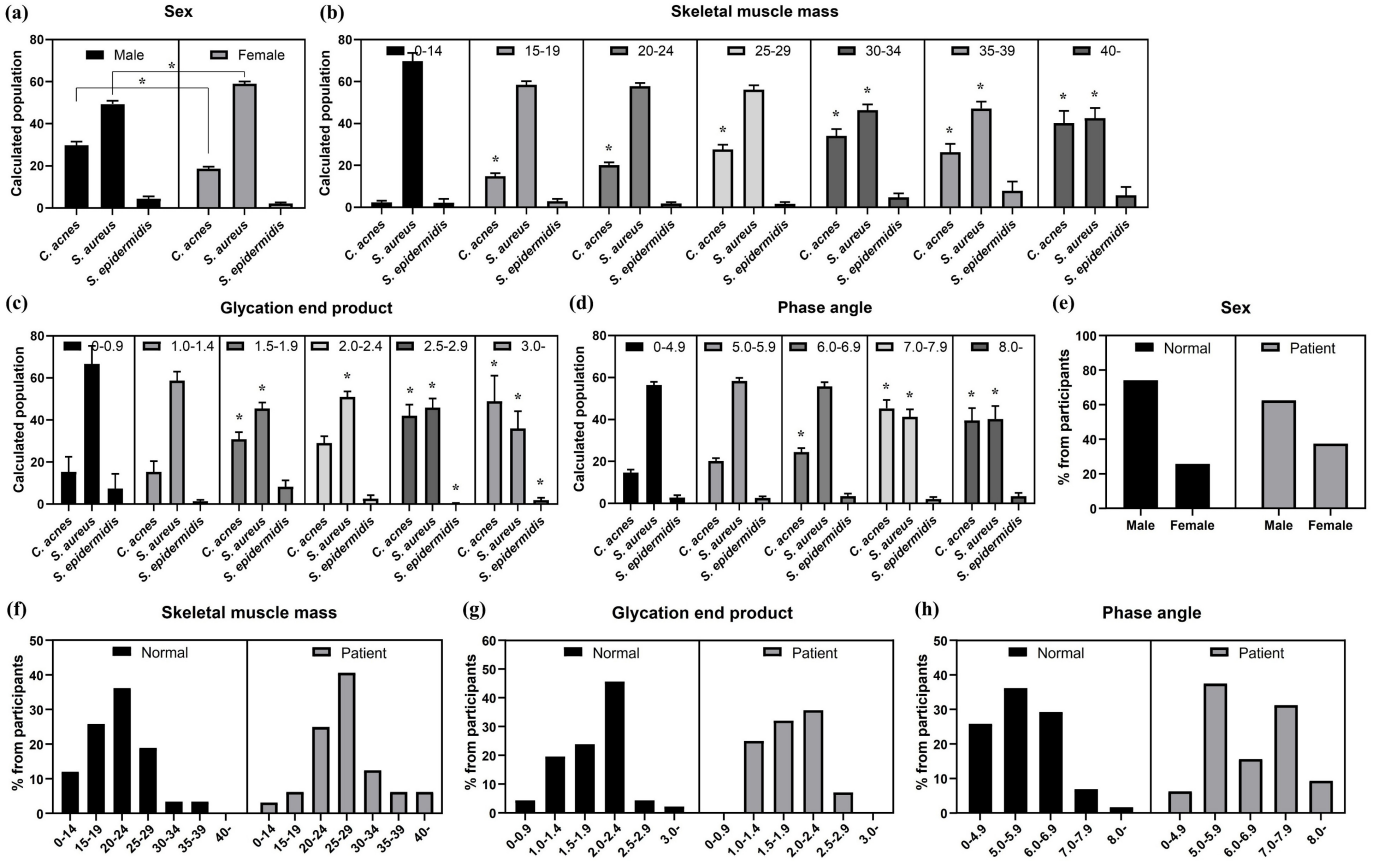
The analysis of skin microbiota composition in different participant subgroups based on body composition analysis its comparison between the normal and patient groups. **(a)** Skin microbiota composition by sex. **(b)** Skin microbiota composition by skeletal muscle mass. **(c)** Skin microbiota composition by advanced glycation end products. **(d)** Skin microbiota composition by phase angle. **(e)** Sex distribution between acne vulgaris patients and normal groups. **(f)** Skeletal muscle mass between acne vulgaris patients and normal groups. **(g)** Advanced glycation end products between acne vulgaris patients and normal groups. **(h)** Phase angle between acne vulgaris patients and normal groups. (*: *p*-value < 0.05).

Sex significantly affected bacterial abundance, with males having higher *C. acnes* and *S. epidermidis* abundance and lower *S. aureus* abundance (Figure 5a). Higher skeletal muscle mass was generally associated with higher *C. acnes* and *S. epidermidis* abundance, and lower *S. aureus* abundance (Figure 5b). Higher advanced glycation end products and phase angle were associated with higher *C. acnes* abundance and lower *S. aureus* abundance (Figures 5c, d).

Statistical analysis revealed demographic and physiological differences. The normal group was more male-dominated (74.1%), whereas the patient group had a more balanced sex ratio (Figure 5e). The normal group had lower skeletal muscle mass, peaking at 20–24 (36.2%), whereas the patient group had significantly higher muscle mass, peaking at 25–29 (40.6%) (Figure 5f). The patients had slightly higher and more clustered advanced glycation end products compared to wider and lower ranges in the normal group (Figure 5g). The patients also tended to have higher phase angle values (Figure 5h).

### 3.5 Skin microbiota composition according to computed body composition

**Figure 6:**
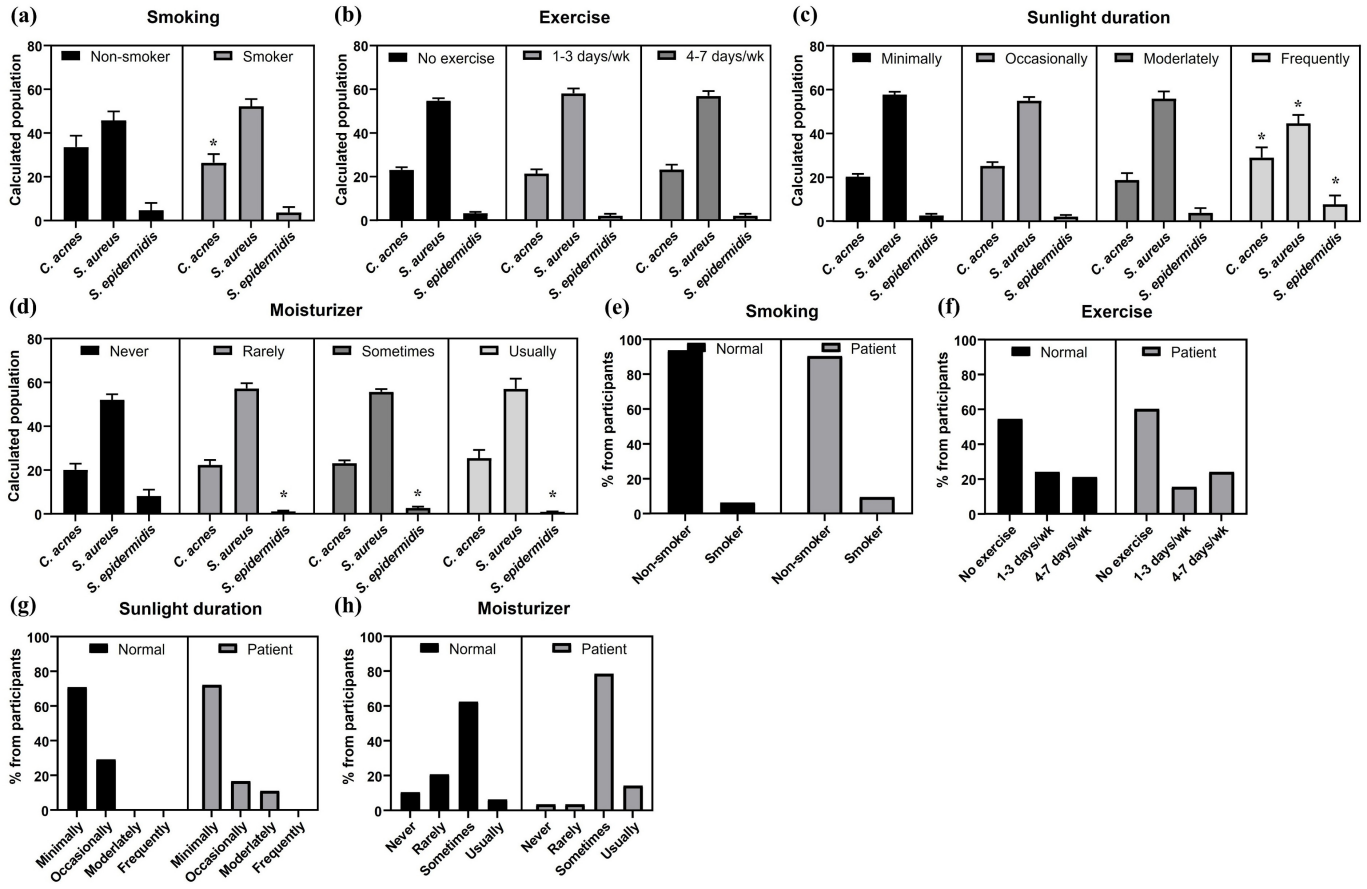
The analysis of skin microbiota composition in different participant subgroups based on computed body composition analysis its comparison between the normal and patient groups. **(a)** Skin microbiota composition by body fat percentage. **(b)** Skin microbiota composition by ECW percentage. **(c)** Skin microbiota composition by BMI. **(d)** Skin microbiota composition by WHR. **(e)** Skin microbiota composition by SMI. **(f)** Body fat percentage between acne vulgaris patients and normal groups. **(g)** ECW ratio between acne vulgaris patients and normal groups. **(h)** BMI between acne vulgaris patients and normal groups. **(i)** WHR between acne vulgaris patients and normal groups. **(j)** SMI between acne vulgaris patients and normal groups. (*: *p*-value < 0.05).

Computed body composition analysis showed a correlation with bacterial abundance. Body fat percentage showed an inverse correlation with *C. acnes* and a positive correlation with *S. aureus* (Figure 6a). ECW ratio also showed this trend (Figure 6b). A high SMI was associated with higher *C. acnes* and *S. epidermidis* abundance, and lower *S. aureus* abundance (Figure 6e). BMI and WHR showed minimal effect on bacterial composition (Figures 6c, d).

Body composition analysis showed that patients tended to have a higher overall body fat percentage. The distribution of body fat percentage differed between groups (Figure 6f). While the normal group was mainly concentrated in the range of 20%−29%, the patient group was more evenly distributed with a significantly higher proportion of patients exceeding this range. Similar but more subtle trends were observed in BMI, SMI, and WHR (Figures 6g, h, i). The opposite trend was observed for ECW, with 78.1% of patients belonging to the group with the lowest body fat percentage (Figure 6j).

## 4 Discussion

The skin microbiome, a complex ecosystem that inhabits human skin, is essential for maintaining both skin health and overall wellbeing (31). The composition of this microbial community is associated with a variety of host factors, including lifestyle choices, physiological parameters, and body composition (19–21).

This study uncovered significant microbiological differences between individuals with acne vulgaris and healthy controls. We observed an elevated abundance of *C. acnes* in patients, consistent with its known pathogenic role (32, 33). However, the wide variation suggests that other factors, such as specific *C. acnes* strains or the host's immune response, also play a part. Interestingly, the protective commensal bacterium *S. epidermidis* was significantly reduced in acne patients (34, 35). This reduction suggests a potential impairment of its protective functions, such as suppressing growth of *C. acnes* or influencing anti-inflammatory effects (36, 37). Conversely, *S. aureus* abundance was lower in patients, suggesting that this bacterium may potentially play a protective role in certain circumstances (10). These findings challenge the conventional view of *S. aureus* as a mere opportunistic pathogen.

Regarding associations with daily life, smoking showed a slight positive correlation with *S. aureus*, a bacterium associated with skin inflammation (38, 39), but the overall effect was smaller than in previous studies (19, 40). Surprisingly, sun exposure was correlated with *S. epidermidis*. It is possible that within a certain range, UV light can promote the growth of beneficial bacteria such as *S. epidermidis* (41). While moisturizer use did not significantly alter microbial abundance, the microbiome of acne patients still differed from that of the normal group. It is also noteworthy that no significant correlation was found between exercise frequency and bacterial communities, suggesting that within the limited microbial scope of our analysis, other unexamined bacterial species might be involved in the effects of exercise on the skin microbiome.

Correlations between the skin microbiota and clinical parameters were observed. *C. acnes* abundance was correlated with menstruation, likely due to hormonal changes in sebum secretion (41–43). Increased skin sensitivity in patients was associated with inflammation. Weak correlations between bacterial abundance and skin conditions such as freckles or pigmentation suggest subtle connections between these conditions and the skin microbiota (44). Body composition was significantly associated with the skin microbiome. Increased muscle mass and decreased body fat were associated with *C. acnes* and *S. epidermidis* and decreased *S. aureus*, suggesting a link between metabolism and skin health. The association between ECW ratio and microbiome further supports this hypothesis, given that ECW reflects hydration status and cellular health (45).

Analysis of body composition data provided new insights into the relationship between physiological parameters and the skin microbiota. A notable finding was that male had significantly higher levels of *C. acnes* and *S. epidermidis* and lower levels of *S. aureus* compared to female, likely due to hormonal or physiological differences (19). The correlation between muscle mass and SMI suggested a potential link between muscle metabolism and skin health. Although the underlying mechanism remains unknown, factors secreted by muscle tissue might influence the composition of skin microbiota. Body fat percentage showed an inverse correlation with *C. acnes* and a positive correlation with *S. aureus*, which could be due to differences in sebum production or other physiological factors associated with body fat. A similar trend was observed in the ECW ratio, which reflects the balance of extracellular and intracellular water, suggesting a potential link between fluid homeostasis and the skin microbiota (45). BMI and WHR had minimal effects on the microbiota, suggesting that these factors did not substantially alter the skin microbiome.

Our results also provide further context of physiological features and the skin condition. For example, the results measured using Systemic Immune-Inflammation Index (SII) and Systemic Inflammation Response Index (SIRI), is positively correlated with psoriasis (46). These findings support our observation that systemic evaluation on physiological state using body composition analyzer can be related to skin health and disease. Similarly, other study demonstrated a strong association between the skin microbiome and biophysical characteristics, suggesting that a broad approach combining microbial and physiological data can lead to a more accurate classification of skin types and aging groups (47). This also supports our finding that the skin microbiome is deeply related with the host's overall physiology.

Differences in muscle mass, AGEs, fat mass, and phase angle between patients and controls support the idea that physiological factors can influence skin health (48, 49). The higher muscle mass, and AGEs in patients align with a previous metabolic role in acne vulgaris (50). This is further supported by the higher phase angle observed in the patients (51, 52).

This study underscores the complex relationship between the skin microbiota, acne vulgaris, lifestyle, and host factors. The significant role of *S. epidermidis* in skin health suggests that microbiome restoration therapies could be beneficial for acne treatment. External factors such as sex, body composition, and metabolism were clearly correlated with the skin microbiome and consequently, skin health. These findings suggest the need for personalized acne management approaches that considers both microbial and host factors.

Several limitations should be considered when interpreting these results. The participant cohort included both acne patients and healthy controls, introducing heterogeneity that, while reflective of real-world practice, could be a confounding factor. Additionally, the study design aimed at investigating skin microbiota and acne patients within the general population resulted in a relatively low proportion of participants diagnosed with clinical acne vulgaris. Future research should focus on more homogeneous groups of acne patients across different severity levels to clarify the role of specific microbes in various acne symptoms.

## Data Availability

The original contributions presented in the study are included in the article/supplementary material, further inquiries can be directed to the corresponding author.
